# DNA methylation impact on Fabry disease

**DOI:** 10.1186/s13148-021-01019-3

**Published:** 2021-02-02

**Authors:** Teodolinda Di Risi, Roberta Vinciguerra, Mariella Cuomo, Rosa Della Monica, Eleonora Riccio, Sirio Cocozza, Massimo Imbriaco, Giovanni Duro, Antonio Pisani, Lorenzo Chiariotti

**Affiliations:** 1 CEINGE - Biotecnologie Avanzate, Via Gaetano Salvatore, 486, 80145 Naples, Italy; 2 Department of Public Health, University Federico II of Naples, Via S. Pansini, 5, 80131 Naples, Italy; 3 Department of Molecular Medicine and Medical Biotechnology, University Federico II of Naples, Via S. Pansini, 5, 80131 Naples, Italy; 4 Institute for Biomedical Research and Innovation, National Research Council of Italy (IRIB CNR), Palermo, Italy; 5 Department of Advanced Biomedical Sciences, University Federico II of Naples, Via S. Pansini, 5, 80131 Naples, Italy

## Abstract

**Background:**

Fabry disease (FD) is a rare X-linked disease caused by mutations in GLA gene with consequent lysosomal accumulation of globotriaosylceramide (Gb3). Women with FD often show highly heterogeneous symptoms that can manifest from mild to severe phenotype.

**Main body:**

The phenotypic variability of the clinical manifestations in heterozygous women with FD mainly depends on the degree and direction of inactivation of the X chromosome. Classical approaches to measure XCI skewness might be not sufficient to explain disease manifestation in women. In addition to unbalanced XCI, allele-specific DNA methylation at promoter of GLA gene may influence the expression levels of the mutated allele, thus impacting the onset and the outcome of FD. In this regard, analyses of DNA methylation at GLA promoter, performed by approaches allowing distinction between mutated and non-mutated allele, may be much more informative. The aim of this review is to critically evaluate recent literature articles addressing the potential role of DNA methylation in the context of FD. Although up to date relatively few works have addressed this point, reviewing all pertinent studies may help to evaluate the importance of DNA methylation analysis in FD and to develop new research and technologies aimed to predict whether the carrier females will develop symptoms.

**Conclusions:**

Relatively few studies have addressed the complexity of DNA methylation landscape in FD that remains poorly investigated. The hope for the future is that ad hoc and ultradeep methylation analyses of GLA gene will provide epigenetic signatures able to predict whether pre-symptomatic female carriers will develop symptoms thus helping timely interventions.

## Background

Fabry disease (FD) is a metabolic, lysosomal storage disorder (LSD) caused by deficient lysosomal alpha-galactosidase A (α-GAL A) activity consequent to mutations in the GLA gene (Xq21.3-q22) encoding the alpha-galactosidase enzyme [1–3].

*Biochemical and clinical aspects*: The enzyme deficiency alters the metabolism of some glycosphingolipids, mainly globotriaosylceramide (Gb3) and Lyso-Gb3 (deacetylated form), causing their storage in lysosomes of various cellular types, mainly cells of vascular endothelium [1]. Gb3 accumulation is responsible for the systemic clinical manifestations and the renal, cardiac and cerebrovascular complications, or a combination of them, which can lead, between the fourth and fifth decade of life, to a premature death [2].

*Epidemiology:* The disease incidence is about 1 in 117 000 live births for males [4], although recent newborn screening surveys suggest that the incidence may be much higher, up to 1:3100 [5–7]. The nonspecific nature of the symptom of Fabry disease and the common occurrence of complications make likely that the prevalence is higher than estimated because many patients remain undiagnosed.

*Diagnosis*: FD is suspected on the basis of clinical and anamnestic-familial data, and it is confirmed by genetic and biochemical assays, specifically identification of one of the several pathogenic gene variants and the evaluation of α-galactosidase A activity that may be null or reduced [8]. The evaluation of substrates of the GLA enzyme, Gb3 and Lyso-Gb3, is used as complementary diagnostic tool [8]. While a first level of screening may be performed by biochemical analysis in males, genetic analysis is required in women because of the unreliability of α-galactosidase A activity test in females [4–8].

*Current Therapies:* The availability of effective therapies, including the recombinant Enzyme Replacement Therapy (ERT), has had an important impact on the management of the affected patients' clinic, ameliorating the prognosis and quality of life. The data available in the literature emphasize the importance of a specific therapeutic intervention as early as possible, before organ involvement becomes irreversible. A precise and timely diagnosis is essential for an early ERT start which is able to stop or slow the progression of the disease, thus improving their quality of the patients’ life [9–14].

*Gender differences:* although FD was classically considered an X-linked recessive disorders, the frequency of clinical manifestations in women turned out to be so high that to date the term “recessive” is considered inappropriate. The most common symptoms observed in Fabry disease, such as neuropathic pain, vascular skin lesion, sweating abnormalities, temperature intolerance and proteinuria, occur in males earlier, in a more severe fashion and progress faster than in females [4–7]. The high variability of clinical manifestations among women, even if sharing a specific genetic variant, is thought to be related to the degree and direction of X chromosome inactivation (XCI) that may favor the mutant allele in variable manners in different individuals and in different tissues of an individual patient. However, data reporting XCI-skewness in female with different clinical phenotype showed the conflicting results some supporting [15–18] and some not supporting [19–21] correlation between XCI and clinical features. A possible explanation of such discrepancies is the emerging dynamic nature and the complexity of XCI phenomenon, much higher than previously believed. Such complexity includes, among other, the phenomenon of XCI escape, tissue-specific and cell-to-cell differences of XCI process, the intricated, gene-specific and gene region-specific role of DNA methylation exerted on both active X (Xa) and inactive X (Xi) chromosomes [22]. It is thus likely that traditional tests evaluating XCI degree and direction are not sufficient and more sophisticated technical approaches and bioinformatic analyses should be applied in order to explain different clinical manifestations, eventual unbalanced expression of normal and mutant alleles and, possibly, to predict the evolution of the disease in women.

*Aim:* The purpose of this review is to summarize and critically evaluate the published works addressing the potential role of DNA methylation in the variability of clinical manifestations and in the early diagnosis of FD in female patients. All the papers found in Pubmed, using as keywords “DNA methylation” and “Fabry Disease,” addressing this subject are discussed in this Review. We highlighted the methodological aspects and possible suggestions for future methylation analyses that can be applied to FD and possibly extended to other X-linked diseases. We believe that one of the main objective of advanced DNA methylation studies in FD should be to gain the ability to predict the onset and evolution of the disease in women carriers allowing timely therapeutic interventions.

## X-inactivation and gender differences in Fabry disease

Fabry disease is characterized by genotypic heterogeneity and phenotypic variability. Phenotypic heterogeneity is considerable, with two main forms, classical and late onset disease [23, 24]. The assessment of genotype–phenotype associations in Fabry disease is complicated by several factors including the rarity of the disease, the considerable allelic heterogeneity (more than 1000 GLA variants described), the variation in clinical expressiveness and the scarcity of published clinical data [23].

Males with pathogenic GLA mutations have practically no residual α-Gal activity and develop classical FD with the onset of symptoms (dysesthesia, gastrointestinal disorders, angiokeratomas, autonomous dysfunction) in childhood and, as we age, the risk of developing life-threatening complications involving vital organs, including progressive renal failure, stroke and hypertrophic cardiomyopathy with myocardial fibrosis and arrhythmias is increasing [25–29]. The variability of clinical symptoms in the female population is likely related to the modalities of X-inactivation. Yet in 1996 Redonnet-Vernhet et al. [15] described twin sister females discordant for Fabry disease, where one showed symptoms of the disease and the other did not [15] and speculated that this was due to differentially balanced X-inactivation. In addition to the differences in symptoms between carrier females affected by FD, tissues-specific differences in terms of X-inactivation may be present in the same individual [18]. This event may be related to the cell-specific inactivation of X chromosome. As a consequence, FD can present variable damage at different organs in females bearing the same mutations. For the same reason, the measurement of α-Gal activity in plasma or leukocytes, the reference method for laboratory confirmation of diagnosis in male patients, is often inconclusive in female patients who may have enzymatic activities ranging from low to normal values [18].

## DNA methylation and X-inactivation

DNA methylation is among the most studied epigenetic modifications that involve the transfer of a methyl group to the fifth position of a cytosine nucleotide. Over the past two decades, DNA methylation studies have fueled more interest due to its role in repressing gene expression and in regulating many cellular processes. These include embryonic development, transcription, chromatin structure, X chromosome inactivation, genomic imprinting and chromosome stability [30, 31]. Aberrant DNA methylation profiles have been detected in several diseases, including cancer and genetic disorders [32, 33]. DNA methylation analysis gave an essential contribution to shed light on the complex epigenetic regulation of X chromosome genes [34–36]. It is widely described that the inactive X chromosome in female mammalian cells is highly methylated [37, 38] while the active one is not. However, the X chromosome epigenetic landscape may be more complex than previously believed being the gene bodies and intergenic regions often highly methylated on Xa [22]. Moreover, several studies used DNA methylation to identify genes that escape from inactivation and to highlight active-X-specific DNA methylation at particular loci [39]. Different methods have been also developed to assess the skewness of X-inactivation, based on the allele differential methylation at polymorphic regions [40]. The currently most used method to evaluate the skewness of X-inactivation, the HUMARA test, is based on the analysis of the methylation state in the polymorphic region of the human androgen receptor gene (AR) [41]. HUMARA is based on digestion by enzymes sensitive to the methylation state that digest the specific region when it is not methylated. Restriction enzymes are able to cut only the active chromosome leaving the inactive chromosome intact. Therefore, the amplification of the AR locus takes place only on the allele of the inactive X chromosome, left intact by the enzymatic cut. The origin of each allele is then eventually recognized on agarose gel because of a likely differential length due to a highly polymorphic CAG repeat region in AR exon 1 opportunely included in the amplicon. However, this approach is based on the assumption that the active X (X*a*) and inactive X (X*i*) chromosomes are uniformly active or inactive, respectively. However, recent evidence shows that this assumption may be erroneous because the diverse X chromosome regions may behave differently [34]. In addition, the phenomenon of XCI escape, according to which some genes can escape inactivation, can vary considerably not only between different individuals but also within each individual, including tissues and even between individual cells within the same tissue [42, 43]. In FD, the study of DNA methylation has been proposed as a possible means of obtaining clarifications on the mechanisms underlying the complexity of FD. In this review, we will discuss studies investigating the role of DNA methylation mechanism in Fabry disease to explain some peculiarity of FD clinical manifestation such as phenotypic variability in women.

## DNA Methylation studies in patients with Fabry disease

In this section our attempt was to include all the articles found in Pubmed (keywords: Fabry Disease and DNA methylation) addressing the role of DNA methylation in FD. In particular we picked all articles studying the methylation at GLA gene in FD patients while only a selection of recent papers addressing in general the relationship between unbalanced XCI and severity of Fabry disease were here considered (see also Table 1). As mentioned above, the results from recent studies pointed out how the analysis of DNA methylation may help to explain the variability of clinical phenotype observed in Fabry disease (FD). DNA methylation occurs at both X and autosomal genes, prevalently at cytosine residues of CpG sites, and has important regulatory effects on gene expression [31]. In this section we will discuss the current knowledge on how DNA methylation may, by different ways, influence Fabry phenotype. A summary of the main studies featured in this review, reporting the sample characteristics, the method used and main findings of each study, is provided in Table 1.

**Table 1:** Summary of the main characteristics of the evaluated studies

References	Samples	Type of sample	Method	Main findings
Redonnet-Vernhet et al*.* [15]	2 FD women	Peripheral blood leukocytes; Fibroblasts	HUMARA assay	Unbalanced XCI in monozygotic twins’ fibroblasts in opposite direction. This is the first documented case of female twins discordant for FD
Maier et al*.* [19]	28 FD women	Blood	HUMARA assay	Fabry heterozygous females showed a random X inactivation and no significant correlation was found between X inactivation patterns and clinical phenotype
Elstein et al*.* [20]	77 FD women	Peripheral blood leukocytes	HUMARA assay	XCI did not correlate with signs and symptoms of classic Fabry disease
Hübner et al*.* [51]	9 FD patients	Peripheral blood leukocytes	CALCR Methylation-specific PCR- High-resolution melting CALCR sequencing	A specific CpG of autosomal CALCR gene is differentially methylated in ERT treated and non-ERT-treated FD patients indicating that this CpG could be an epigenetic biomarker of FD
Echevarria et al*.* [18]	56 FD women	Peripheral blood; Mouth epithelial cells; skin biopsy; Urine	HUMARA assay	XCI significantly impacted the phenotype and natural history of FD in females, supporting the correlation between XCI and clinical phenotype
Hossain et al*.* [44]	4 FD women	Peripheral blood; spinal fluid	GLA Methylation-sensitive restriction enzymes analyses GLA Bisulfite Sanger sequencing	Allele-specific GLA methylation correlated with the severity of FD phenotype
Juchniewicz et al*.* [21]	12 FD women	Saliva	HUMARA assay	XCI pattern did not correlate with Fabry disease severity scores
Hossain et al*.* [46]	36 FD women	Peripheral blood; skin fibroblasts	GLA Methylation-sensitive restriction enzymes analyses GLA Bisulfite Sanger sequencing	Methylation of the GLA non-mutated allele was proportionally correlated with the clinical severity score (FASTEX score)
Yanagisawa et al*.* [45]	4 FD women	Fibroblast from Skin tissue	Allele-specific GLA expression (RT-PCR)	mRNA expression level of the GLA mutant allele correlated with disease severity
De Riso et al*.* [55]	3 FD women	Peripheral blood	High coverage-amplicon bisulfite sequencing (HC-ABS) versus HUMARA	Substantial concordance in direction and entity of the methylation imbalance between AR and GLA genes. Clearly distinct allele-specific epiallele profiles were obtained by epiallele distribution analysis
Rossanti et al*.* [56]	9 FD women	Blood leukocytes; Urine sediments	HUMARA assay GLA Ultra-deep targeted RNA Sequencing	Skewed XCI explained the severity of FD in only limited number of female cases

In 2016, Echevarria L. et al*.* conducted a study whose purpose was to evaluate the presence of skewed XCI in women with Fabry disease, the possible correlation between XCI in different tissues and its contribution to the phenotype [18]. The focus of the study, carried out on a cohort of 56 women with Fabry disease, was the assessment of XCI direction in 4 different tissues. The XCI balance was established by HUMARA test addressing the methylation state at the polymorphic AR exon 1 region. Such analysis, performed by Echevarria et al*.* [18], showed that 71% of the entire sample had a random XCI in the analyzed tissues, the remaining 29% showed a skewed XCI. In this latter group, 6 out of 16 preferentially expressed the wild-type (wt) allele and 10 out of 16 the mutant GLA allele in at least 2 out of 4 of the analyzed tissues. This data indicated that there were no selection mechanisms in favor of the wild-type GLA allele expression. The women in which the non-mutant allele was preferentially repressed showed a higher severity score of disease. The data from Echevarria's group [18] revealed a significant and calculable correlation between the XCI direction observed in the blood with that present in the other tissues considered.

A demonstration of how much the prevalence, due to an unbalanced DNA methylation, in the expression of the mutated alleles for the GLA gene can seriously influence the clinical phenotype, was provided by Hossain et al*.*, in the 2017 [44]. Hossain’s group studied a severe case of a heterozygous female Fabry patient suffering acroparesthesia, facial dysmorphism, left ventricular hypertrophy and intellectual disability in addition to a proven family history particularly relevant to Fabry disease. Biochemical analysis showed the absence of α-gal A activity, massive excretion of Gb3 and Gb2 (galabiosylceramide) in urine in addition to high levels of lyso-Gb3 in dried blood spot (DBS) and plasma. Along with HUMARA analysis, the authors performed methylation analysis of GLA promoter region and were able to distinguish methylation pattern of mutant versus wt alleles. The experimental design was favored by the fact that, in the analyzed case, the pathogenic mutation was located in the GLA exon 1 (c.36C > A) in close proximity of the promoter region. The methylation study was carried out by Sanger sequencing analysis at two CpG sites within HhaI sites one of which included the 36C > A mutation. The analysis was conducted both on bisulfite-treated and untreated genomic DNA. Although conducted by a relatively low-resolution technique, the study by Hossain et al. [44] provided the first evidence of correlation between methylation state of wt GLA allele and the early onset and severity of disease manifestations in one female patient. In support of this observation the authors found in other three relatives of this patient analogous correlation. In fact, the methylation state of the 2 analyzed CpG sites at wt GLA promoter was lower in the asymptomatic or pauci-symptomatic females of the same family [44].

In 2019, the same family was chosen to study how autophagic process dysfunction can affect the different clinical phenotype in heterozygotes females carrying the same mutation [45]. The authors revealed that autophagic flux abnormalities, along with levels of p62 and lysosome morphology, were directly related to severity of symptoms. Moreover, the authors investigated the expression of GLA gene by allele-specific PCR and found that mutant allele was highly expressed in the patient with severe clinical manifestations compared to the sisters with few symptoms, confirming a correlation between e mRNA expression level of the mutant allele and disease severity. Therefore, the authors concluded that in patients with Fabry allele-specific mRNA expression as well as autophagy dysfunction is related to the severity of the disease [45].

To confirm that epigenetics could play an important role in understanding the variability of clinical severity in FD, Hossain et al*.* in 2019 [46] showed a significant correlation between the FASTEX score (severity of the phenotype) and the methylation of the healthy allele along with lyso Gb3 levels. They examined skin biopsies from 36 women heterozygous for GLA mutations and measured the levels of DNA methylation at GLA locus by methylation-sensitive restriction enzyme. They identified a clear and proportional correlation between methylation of the non-mutated allele and the clinical severity score measured by FASTEX. They also found a strong correlation between the severity of the phenotype and lyso-Gb3 accumulation for heterozygous Fabry disease in females. Hence, the authors observed that in heterozygous women with severe phenotype, the non-mutated allele was methylated at higher levels, causing a reduction of the transcription of the “healthy” GLA gene and the lack of synthesis of α-GAL A. This phenomenon ultimately led to the accumulation of lyso-Gb3, with the consequent increased severity of the disease. Based on these considerations, the authors concluded the study of allele-specific DNA methylation may contribute to the determination and understanding of the clinical variability, as well as an early diagnosis that would allow a specific therapy to be started promptly.

Some of the symptoms directly related to FD, such as neuropathic pain, have been related to epigenetic mechanisms [47]. Neuropathic pain is one of the main symptoms that characterizes the early stages of FD and it is drastically reduced after enzyme replacement therapy [48–50]. One of the possible mechanisms that causes such reduction involves the Calcitonin Gene-Related Peptide (CGRP) pathway. CGRP mediates pain transmission through the activation of the calcitonin receptor (CALCR). Hübner et al. [51] carried out a retrospective analysis in patients affected by Fabry and treated with enzyme substitution therapy addressing the methylation status of the autosomal calcitonin receptor promoter region [51]. The authors showed that in ERT-treated patients the methylation in position -78,504 CpG of the CACLR gene, described by authors as part of CALCR promoter region, was higher than in untreated patients. The authors suggested that methylation of CALCR gene could prevent the binding of HIF-1α and consequently, CALCR expression thus possibly inhibiting pain transmission. However, due to the relatively small sample size (9 Fabry patients including 6 non-ERT treated and 3 ERT treated), it was not clear whether the high methylation levels observed at a specific CpG site was related to ERT or instead to severity of disease. In this latter case, methylation at CALCR could be proposed as a biomarker for severity of disease [51].

Recently, our group provided evidence that studying DNA methylation at specific autosomic loci at a single-molecule resolution allows one to analyze cell-to-cell methylation differences in a given cell population and to track possible evolution of methylation profiles during time [52–54]. This was obtained by the so-called “epiallele distribution analysis” based on the concept that different cells with the same origin can be actually considered a mixture of epigenetically heterogeneous cells in which each combination of methyl CpGs at a given locus represents a specific epiallele. The epiallele distribution analysis was performed by high coverage-amplicon bisulfite sequencing (*HC-ABS)* consisting in the frequency determination of each methylated CpG combination among hundred thousands molecules analyzed after bisulfite treatment, amplification of specific loci and high coverage sequencing by next generation methods. Such ultra-deep DNA methylation analysis was subsequently applied to track two X-linked loci, GLA and AR, and to investigate whether females showed allele-specific epiallelic patterns [55]. The peripheral human whole blood from 3 women was analyzed in parallel by HUMARA and High Coverage-Amplicon Bisulfite Sequencing (HC-ABS) approaches targeting AR and GLA genes. By this approach it was possible to investigate the asset of both methylated and unmethylated CpG sites present in each amplicon-derived sequence with high precision. To give a measure of the complexity of such analysis it is useful to consider that, as an example, in a mixed population of cells the analyzed region of AR gene, which included 14 CpG sites, may give origin to 2^14^ (16,384) possible combinations while the analyzed region of GLA gene may generate 2^17^ (131,072). Although the study [55] was carried out on a limited number of subjects, the results highlighted the potential power of ultra-deep analysis to study in the future the events underlying the interindividual phenotypic variations and, hopefully, to predict the tendency to develop clinical symptoms of recessive X-linked diseases in women.

## Discussion and conclusions

To date, few studies investigated the role of DNA methylation in Fabry disease. Most of these studies addressed the relationships between the phenotypic variability of the clinical manifestations in heterozygous women with FD, directions of XCI and the methylation state of GLA gene.

**Fig. 1:**
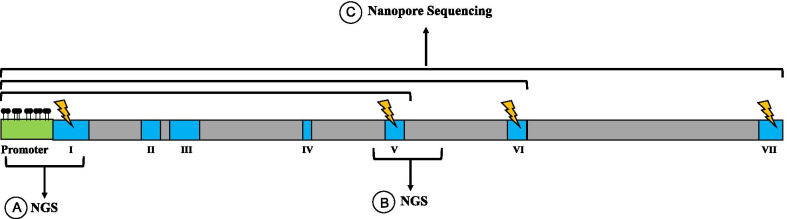
Different possible methods to analyze allele-specific methylation at GLA gene. Schematic representation of GLA gene showing promoter, exons and introns positions (not in scale) and exemplificative positions of hypothetical point mutations. The methylation state at promoter region is the best candidate factor able to regulate GLA gene expression. In order to identify single molecules containing information necessary to distinguish the two alleles and at the same time methylation profiles, different approaches can be used according to specific cases. In the case in which the mutation occurs close to promoter region (e.g., in exon 1), it is possible to perform amplicon bisulfite sequencing (**a**) with amplicon length ranging between 200 and 600 bp and then distinguish alleles by bioinformatic analyses, as performed by Echevarria et al. [16]. If mutation is located at downstream exons (e.g., exon 5), it is possible to analyze allele-specific DNA methylation at a region surrounding mutations that may be not informative for regulation of the gene (**b**), unless in these patients a polymorphisms in the promoter region may be associated with the mutated allele (e.g., following other members of the same family) as shown in De Riso et al. [56]. Alternatively, Nanopore sequencing may analyze much longer regions allowing to match methylation status of the promoter with mutation present in any position of the gene on the same molecule (**c**). Advantages of using this method are that it is possible to perform methylation analyses without PCR amplification and bisulfite treatment. However, it may be necessary to isolate the whole GLA genomic region by in vitro CRISPR Cas9 system [58]

Most of the studies defined the extent and direction of XCI through HUMARA test that, being an inexpensive and fast method, is the most widely used technique to date to evaluate XCI skewness. Independently on the X-linked disease and the related mutant gene under consideration, HUMARA analysis measures the differential methylation state of paternal and maternal alleles at the AR locus. However, because HUMARA analyzes only few CpG sites at only one locus, it does not give information on the complex scenario and the methylation dynamics occurring along the X chromosome, especially at X-linked genes distant from AR gene. In fact, the X-inactivation process has been recently largely revisited revealing a high epigenetic variability among different loci both within inactive and, possibly, active X-chromosomes [22, 34, 43]. It is thus not surprising that XCI skewness in women suffering of Fabry disease, as assessed by traditional HUMARA tests, was found not sufficient to explain different clinical courses in women and that conflicting results were obtained [15–21]. Since the great majority of pathogenic variants lies in GLA exons, a more direct approach to reveal unbalanced expression of mutant and non-mutant alleles may be the quantitative analysis of allele-specific transcripts by RNAseq-based analyses [56]. The limitation of this approach is that it neither gives information about the relative number of cells expressing each allele nor on the mechanisms underlying the eventual unbalanced expression. Moreover, the results can be influenced by post-transcriptional mechanisms. Thus, transcripts analysis may be very useful to “capture” the actual expression of each allele in a given tissue in a given moment but does not provide data useful to predict the eventual evolution of gene expression program over time. This can be better investigated by methylation analysis of GLA alleles. Experiments performed to establish the GLA methylation state, distinguishing mutant and non-mutant alleles, included the use of methylation-sensitive restriction enzymes or bisulfite treatment of genomic DNA followed by PCR amplification of GLA promoter [17, 18, 21, 44–46]. The results of these experiments revealed that the amount of methylation of non-mutant allele correlated with the severity of the disease [55]. Single molecule methylation analysis and epiallele distribution analysis, obtained by high coverage bisulfite sequencing of GLA gene, promise to help to predict the evolution of expression potential overtime. We believe that this could be likely achieved because epiallele distribution profiles were demonstrated to be generated in a very well-orchestrated manner and to follow precise spatiotemporal dependent trajectories [41, 52, 54, 57]. However, the above bisulfite-based mentioned methods have the limitation that the mutation, or traceable polymorphic markers, must lie within the amplicons, and thus, in the proximity of the promoter because in this region, effective DNA methylation changes are likely to occur. In fact, DNA methylation in gene body might be higher at the active GLA allele than at the inactive one, as it was demonstrated for several X-linked genes [22]. This further level of complexity requires further investigation and that more sophisticated techniques be applied. A complete scenario including allele specific methylation profiles along the whole GLA gene may be potentially obtained by innovative techniques, e.g., Nanopore sequencing, which provide long reads, covering the whole GLA gene at single molecule level, and the methylation state of each cytosine without the need of bisulfite treatment. A summary of the methods here discussed to analyze DNA methylation at GLA gene is represented in Fig. 1.

The hope for the future is that ad hoc and ultradeep methylation analyses of GLA gene will provide epigenetic signatures able to predict whether pre-symptomatic female carriers will develop symptoms in order to favor timely therapeutic interventions.

## Data Availability

Not applicable.

## Abbreviations

FD: Fabry disease
α-GAL A: α-Galactosidase A
Gb3: Globotriaosylceramide
ERT: Enzyme replacement therapy
AR: Androgen receptor
XCI: X chromosome inactivation
active X: Xa
inactive X: Xi
wt: Wild-type
HC-ABS: High Coverage-Amplicon Bisulfite Sequencing
DBS: Dried blood spot
Gb2: Galabiosylceramide
FASTEX: Fabry Stabilization Index
CGRP: Calcitonin Gene-Related Peptide
CALCR: Calcitonin Receptor
LSDs: Lysosomal storage disease
